# Effects of evidence-based clinical practice guidelines in cardiovascular health care quality improvements: A systematic review

**DOI:** 10.12688/f1000research.18865.3

**Published:** 2019-10-14

**Authors:** Anggie Ramírez-Morera, Mario Tristan, Juan Carlos Vazquez

**Affiliations:** 1 Cochrane Central America & Caribbean Spanish, IHCAI Foundation, San José, San José, 10904, Costa Rica; 2 Universitat Autònoma de Barcelona, Barcelona, Spain, 08041, Spain; 3 Iberoamerican Cochrane Centre, Barcelona, Spain

**Keywords:** Clinical Practice Guidelines. CPG, effect, health care quality

## Abstract

**Background:** The development of evidence-based clinical practice guidelines (EB-CPGs) has increasing global growth; however, the certainty of impact on patients and health systems, as well as the magnitude of the impact, is not apparent. The objective of this systematic review was to assess the effectiveness of the application of EB-CPGs for the improvement of the quality of health care in three dimensions: structure, process and results in the patient for the management of cardiovascular disease.

**Methods:** We followed the methods described by the Cochrane Handbook and present a descriptive analysis because of the high heterogeneity found across the included studies. We searched the Cochrane Central Register of Controlled Trials, MEDLINE and EMBASE databases, as well as the grey literature, between 1990 and June 2016. No language restrictions were applied. Only randomised clinical trials (RCTs) were selected. Three authors independently carried out the data extraction, using a modified version of the Cochrane Effective Practice and Organization of Care form.

**Results:** Of the total of 84 interventions included in the nine RCTs evaluated, three (4%) were related to health care structure, 54 (64%) to the health care delivery process and 27 (32%) to patient outcomes. Regarding the impact of using the EB-CPGs, in 55 interventions (65%), there were no significant differences between control and experimental groups. In four interventions (5%), the result favoured the control group, and the result favoured the intervention group on 25 of the interventions (30%).

**Conclusions:** This systematic review showed that EB-CPGs could be useful to improve the process and structure of health care and, to a lesser extent, to improve the patients’ outcomes. After analysing many studies, we could have one more hypothesis for further research, which could shed more light upon those undiscovered variables that might interfere with the use of the EB-CPGs.

**Registration:** PROSPERO CRD42013003589

## Introduction

This review refers to the changes in the quality of healthcare services that are direct consequences of the systematic use of evidence-based clinical practice guidelines (EB-CPGs). “Evidence-based” means that the recommendations are created using rigorous, unbiased and transparent methods of collation and appraisal, alsousing scientific findings of the highest quality and value to assist in providing optimal clinical care to the patient. ( Guyatt, 1992; Sackett *et al*., 1996). EB-GPCs are evidence summaries and include systematically developed recommendations to assist physicians and patients in the process of making decisions ( Alonso-Coello *et al*., 2010; Glasziou *et al*., 2011). EBCPGs are one of the most available and widely used tools for translating the knowledge generated by scientific research to practice. They should be based on the best scientific evidence available in order to improve the quality of patient care ( Alonso-Coello *et al*., 2010; Grimshaw & Russell, 1993). Experts, institutions, and organizations worldwide have developed EBCPGs in all areas of medicine as a key tool to improve the quality of care Donabedian, 1988; Glasziou *et al*., 2011; Lugtenberg *et al*., 2009; Moore *et al*., 2015 states that before evaluating health care, we must first decide how to define “quality” and whether it depends only on the actions of physicians or if it also depends on contributions from the patients and the healthcare system.

Defining quality is challenging since it is not easy to characterise coherently and objectively. Health must be analysed from a holistic point of view, and guideline developers must determine the ideal amount of influence health should receive from individual preferences and social components. We must also understand the relationship between structural characteristics and healthcare processes, as well as their results in health services ( Donabedian, 1988; Moore *et al*., 2015).

Since the 1990s, increasing numbers of EB-CPGs are being developed. However, it is unknown whether these high-quality recommendations have a beneficial impact on patient health. Despite the high number of recently published EB-CPGs, there are few studies on their effectiveness in improving clinical outcomes, the process and structure quality throughout the healthcare system.

After an exhaustive search, only two systematic reviews (SR) we found on this topic ( Lugtenberg *et al*., 2009; Worrall *et al*., 1997). Worrall *et al*., 1997 only analysed patient outcomes missing two of the proposed Donadebian Model. Lugtemberg 2009 used the full Donabedian (1988) model only including studies from The Netherlands.

This review sought to assess whether the quality of health care improves in patients with the cardiovascular disease when using EB-CPGs vs standard professional medical practice. The primary aim was to assess the impact of the EB-CPGs for the management of cardiovascular diseases on healthcare quality, in terms of patient outcomes, management process, and healthcare structure.

## Methods

We designed a methodology aimed to find and analyse studies measuring the impact of EB-CPGs on the improvement of quality in health care services in the three areas proposed by Donabedian ( Donabedian, 1988; Moore *et al*., 2015): structure, process, and patient outcomes. From the very beginning of the process when the authors wrote this review protocol, it was clear that these items would not be easy to measure and, as the search and data extraction moved forward, it became harder to synthesise the information delivered by the different studies included. The main obstacle was the inconsistency observed in the different outcome measures used by the studies, which included continuous as well as dichotomous values for different clinical conditions and interventions. The intervention definition read as any planned action taken to modify the clinical practice and use of the clinical guidelines for influencing in the clinical practice. The authors followed the methodological recommendations described in the Cochrane Handbook ( Higgins & Green, 2012). This review is registered with PROSPERO (ID: CRD42013003589).

### Study searches

We did a systematic search using the following electronic databases for primary studies (randomised controlled trials (RCTs)): Cochrane Central Register of Controlled Trials (CENTRAL). The Cochrane Library, including the Cochrane EPOC (Cochrane Effective Practice and Organization of Care) specialised database; MEDLINE; EMBASE; CINAHL; PsycINFO; LILACS; Health Technology Assessment Databases and Web of Science, Science Citation Index, and Social Sciences Citation Index.

The review authors combined search strategy for indexed terms and developed free text terms. We included searches of grey literature in different sources, such as reports of the world and regional conferences, academic theses and scientific reports not published in indexed journals. We searched for studies published between January 1990 and June 2016, without any language restriction. An advanced search strategy is available as *Extended data*, Appendix 1 ( Ramirez *et al*., 2019).

### Study selection

We analysed the studies found through the search strategy, and two authors independently selected the articles according to the following inclusion criteria: RCT measuring the change in health care when using EB-CPGs on cardiovascular disease. The study should measure the change in any of the three health care dimensions (structure, process, and patient outcomes).

### Data extraction

Three authors independently performed data extraction using a modified shorter version of the Cochrane Collaboration EPOC Data Collection Checklist translated into Spanish. Apart from preparing the Spanish version, we eliminated several items not applicable to this review, given that we only included RCTs. We used a standardised digital form for data extraction and analysis. We used a standardised digital form for data extraction and analysis. We used Review Manager software (RevMan 5.3) for the data analysis.

We assessed the risk of bias (quality) according to the Cochrane Handbook ( Higgins & Green, 2012). We found very high variability, so the study results were introduced as a narrative in the Results.

Because of the variability between the measurements of the effect of the impact of EB-CPGs on the change of quality in the studies included in this review, it was not possible or appropriate to perform a meta-analysis; therefore, it was not possible to measure the statistical heterogeneity.

## Results

### Study identification and selection

When the first version of the protocol of this Systematic Review was initiated, consider the inclusion of several clinical topics in a single systematic review. We decided to create a series of three systematic reviews on different clinical topics: Cardiovascular Health, Breast Cancer and Child and Mother Health. The initial searches included all those topics with the same inclusion criteria described. After removing duplicates, the search produced 4279 potential studies. After screening by title and abstract, 4051 were excluded. After the full-text evaluation, 96 studies of all clinical subjects were selected, extended data, Appendix 2 ( Ramirez *et al*., 2019). 

### Study characteristics

**Figure 1.**
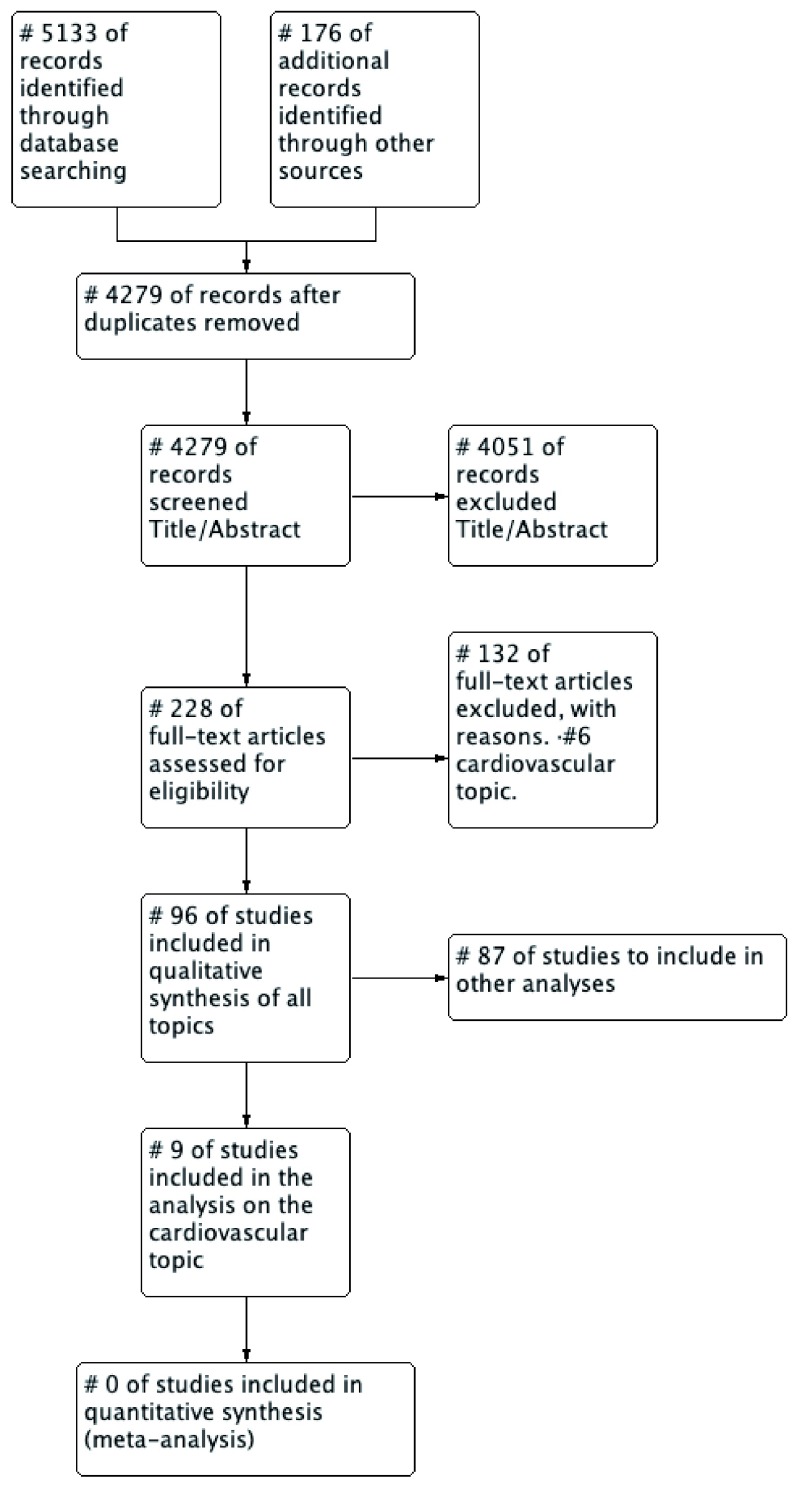
Flow chart of the studies (PRISMA, Moher 2009).

For the analysis, we organised the studies according to the topic or pathology being the core subject of the EB-CPG, and for this report, we only selected RCTs on cardiovascular diseases, because this theme accounts for the higher number of original articles. In total, we selected nine RCTs ( Figure 1). Characteristics of the included studies are available as Extended data, Appendix 2 ( Ramirez *et al*., 2019).

We included nine RCTs analysing cardiovascular diseases ( Beaulieu *et al*., 2004; Berner *et al*., 2003; Ellis *et al*., 2000; Guadagnoli *et al*., 2004; Hand *et al*., 2014; Jäntti *et al*., 2007; Kiessling & Henriksson, 2002; Tierney *et al*., 2003; Tsuyuki *et al*., 2015). All trials were carried out between 2000 and 2015, five in the United States of America, two in Canada, one in Finland and one in Sweden. Eight articles addressed outpatient care and three inpatient care. The EB-CPGs included in the selected trial looked at the following clinical problems: management of stable angina pectoris (over 65 years), unstable angina, dyslipidaemia, acute infarction, heart failure and blood pressure. Besides, the trials included perioperative cardiac evaluations of patients with non-cardiac surgery, cardiopulmonary resuscitation and secondary prevention in patients having coronary artery disease.

A number of the 4,279 studies found in the initial search were not randomised clinical trials, as the authors described in their titles or abstracts. When assessing the full-text articles, we found that many were cluster trials and observational studies with a “before and after” design.

### Quality of evidence assessment

As for the quality of the evidence, we observed the presence of a high or unclear risk of bias for allocation concealment (selection bias), blinding of participants and personnel (performance bias), and blinding of outcome assessment (detection bias), which can be explained by the nature of the interventions studied. We found several types of systematic errors: random sequence generation (selection bias), incomplete outcome data (attrition bias) and selective reporting (reporting bias). We found that the interventions measured yielded outcomes assessed with moderate to low evidence certainty according to the GRADE classification.

### Risk of bias assessment

**Figure 2.**
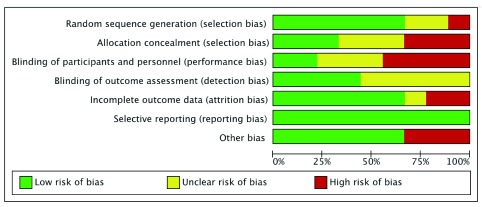
Overall bias risk chart of all included studies.

Analysing the risk of bias in the nine included RCTs studies found the random sequence generation (selection bias) assessment had a low risk of bias (between 50–75%) for allocation concealment (selection bias) 33% low, 33% unknown and 33% high risk of bias. The blinding of participants and personnel (performance bias) obtained almost 50% high risk of bias. The blinding of outcome assessments (detection bias) obtained a 50% low and 50% uncertain risk of bias. Incomplete outcome data (attrition bias) obtained between a 50 and 75% low risk of bias. For the selective reporting (reporting bias), there was a 100% low risk of bias. Finally, other types of bias had a low risk of between 50 and 75% ( Figure 2).

The studies with the lowest and highest risk of bias ( Kiessling & Henriksson, 2002 and Jäntti *et al.*, 2007, respectively) were chosen to be evaluated with the GRADE methodology and to build a summary of findings table. The aim of doing this GRADE table was obtaining the rank of possible grades of the evidence certainty found in the 84 interventions from the nine studies included. The results of these studies produced moderate-to-low evidence certainty, according to the GRADE classification (see Table 1 and Table 2).

**Table 1.** Evaluation of the Certainty of the Evidence for the Jäntti 2007 study on cardiopulmonary resuscitation, according to the GRADE classification.

Are the recommendations presented in the ERC 2005 CPG better than those presented in the ERC 2000 CPG more adequate to improve the outcomes for cardiopulmonary resuscitation?						
Patient or population: Manikins simulation of cardiopulmonary arrest Intervention: CPG ERC 2005 Control: CPG ERC 2000 Bibliography: Jäntti H1, Kuisma M, Uusaro A.. The effects of changes to the ERC resuscitation guidelines on no flow time and cardiopulmonary resuscitation quality: a randomised controlled study on manikins. Resuscitation November 2007;2(75):338–44. [PubMed: 17628319]						
Outcomes	**Average duration** **(seconds ± SE)**		Relative effect (p)	№ of participants (studies)	Certainty of the evidence (GRADE)	Relevance
	**Average with** **ERC 2000**	**Average with** **ERC 2005**				
Total time without flow (s)	**393 ± 19**	**190 ± 23**	**p < 0.001**	34 Control Group: 17 Intervention Group: 17 (1 RCT (Randomized Controlled Trial)	⨁⨁⨁◯ MODERATE ^1, 2^	Critical
Delay to start CPR (s)	**8 ± 6**	**8 ± 4**	p = 0.949	34 Control Group: 17 Intervention Group: 17 (1 RCT (Randomized Controlled Trial)	⨁⨁◯◯ LOW ^1, 3^	Critical
**ERC:** European Resuscitation Council						
**SE:** Standard deviation						
**p:** significance						
**GRADE Working Group grades of evidence**						
**High certainty:** We are very confident that the true effect lies close to that of the estimate of the effect						
**Moderate certainty:** We are moderately confident in the effect estimate: The true effect is likely to be close to the estimate of the effect, but there is a possibility that it is substantially different						
**Low certainty:** Our confidence in the effect estimate is limited: The true effect may be substantially different from the estimate of the effect						
**Very low certainty:** We have very little confidence in the effect estimate: The true effect is likely to be substantially different from the estimate of effect						

**Explanations** 1. For the generation of the random sequence (selection bias) the risk is High: sealed, numbered and opaque envelopes were used for the randomisation of the cases to be treated. It is not clear why people with less work experience or no academic degree were assigned to the ERC 2000. For the allocation concealment (selection bias) the risk is High: It is likely that by the nature of the study the allocation concealment of the selection could not be made. For blinding of participants and staff (performance bias) the risk is High: It is likely that the nature of the study could not prevent participants from knowing which group they belonged to (which CPG they were using). For blinding of the outcome evaluation (detection bias) the risk is Low: A computer automatically collected it. For incomplete results data (attrition bias) the risk is Low: There was no loss of follow-up since it was a single session. For the particular report (notification bias) the risk is Low: A computer automatically collected it. In other risks of bias, the risk of bias is High: The description of the study design was not clear; therefore, we assumed that the study has a high risk of bias.

2. P < 0.001

3. P 0.949. Downgraded -1 for imprecision.

**Table 2.** Evaluation of the Certainty of the Evidence for the secondary prevention of coronary artery disease according to the GRADE classification: ( Kiessling & Henriksson, 2002) study.

Is the active implementation more effective compared to passive implementation as it was used in the control group to improve secondary prevention of coronary artery disease?						
Patient or population: person with coronary artery disease. Intervention: Active implementation (general practitioners participated in learning dialogues using the CPG on secondary prevention of coronary artery disease, method of recurrent cases in their primary care centers) Control: Passive implementation (the CPG was mailed to general practitioners and presented at a conference) Bibliography: Kiessling A1, Henriksson P. Efficacy of case method learning in general practice for secondary prevention in patients with coronary artery disease: randomised controlled study. BMJ October 2002;7369(325):877-80. [DOI: 12386042]						
Outcomes	**Average Change of** **active versus passive** **implementation of the CPG (%)** **(95% CI)**		Relative effect (p)	№ of participants (studies)	Certainty of the evidence (GRADE)	Relevance
	**Control** **Group**	**Intervention** **Group**				
Difference in percentage of LDL change at 2 years [Mean (mmol / L)]	0.7% (CI -4.1 a 5.9)	-9.3 % (CI -15.8 a -2.9)	p < 0.05	176 Control Group: 88 Intervention Group: 88 (1 RCT (Randomized Controlled Trial)	⨁⨁⨁◯ MODERATE ^1, 2^	IMPORTANT
Difference in percent change in total cholesterol at 2 years [Mean (mmol / L)]	1.8% (CI -2.2 a 5.9)	-6.0 % (CI -10.4 a -1.5)	p > 0.05	176 Control Group: 88 Intervention Group: 88 (1 RCT (Randomized Controlled Trial)	⨁⨁◯◯ LOW ^1, 3^	IMPORTANT
**CI:** Confidence interval						
**p:** significance						
**GRADE Working Group grades of evidence**						
**High certainty:**We are very confident that the true effect lies close to that of the estimate of the effect						
**Moderate certainty:**We are moderately confident in the effect estimate: The true effect is likely to be close to the estimate of the effect, but there is a possibility that it is substantially different						
**Low certainty:**Our confidence in the effect estimate is limited: The true effect may be substantially different from the estimate of the effect						
**Very low certainty:**We have very little confidence in the effect estimate: The true effect is likely to be substantially different from the estimate of effect						

**Explanations** 1. For the generation of the random sequence (selection bias) the risk of bias is Uncertain: Not described. For allocation concealment (selection bias) the risk is low: general practitioners and patients did not know in which research group they were assigned. For blinding of participants and staff (performance bias) the risk is low: General practitioners were not aware of being involved in the study and a blinded nurse on which group each patient belonged to, was the one who handled the paperwork, protocols of the investigation and had no contact with general practitioners. For blinding of the outcome assessment (detection bias), the risk is Low: The research codes and databases were not disclosed until the authors completed the statistical analysis. For incomplete results data (attrition bias), the risk is Low: The study used the intention-to-treat analysis and indicated that the follow-up for two years was 86%. For the particular report (notification bias) the risk is Low: The research codes and databases were not disclosed until the authors completed the statistical analysis. For Other biases the risk is Low: None known

2. p < 0.05

3. p > 0.05. Downgraded -1 for imprecision.

### Assessment of study outcomes

The authors grouped the outcomes in simple relative and absolute numbers. There was not a global estimate of the measurements of the effects included in the studies because of the considerable variability of measuring units, as well as the clinical heterogeneity found among the studies included. The measurement of the outcomes reported in the studies was dichotomous, continuous or nominal; the majority were dichotomous and were used to measure the mastery of the process, e.g. the number of patients receiving an adequate treatment following a recommendation versus those who did not.

Of the total of 84 interventions included in the nine RCTs evaluated, three (4%) corresponded to the health care structure dimension, 54 interventions (64%) to the dimension health care delivery and 27 interventions (32%) to the dimension of patient outcomes. Regarding the impact of EB-CPG use, we found that in 55 interventions (65%) there was no significant difference between the control and experimental groups.

In four interventions (5%) the outcome favoured the control group (comparison of the measure of compliance of the recommendations (the average adjusted by the patient characteristics, the setting, and the number of measures applied per patient)). The measure of effects (odds ratio (OR)) regarding the conditions acute myocardial infarction and heart failure, separated by care provided by a cardiologists or primary care physicians, were as follows: acute myocardial infarction, cared by a cardiologist OR 0.81 (95% CI: 0.79 to 0.83), and cared by a primary care physician OR 0.73 (95% CI: 0.71 a 0.76); heart failure, cared by a cardiologist OR 0.88 (95% CI: 0.86 to 0.90), and care by a primary care physician OR 0.79 (95% CI: 0.76 to 0.81).

The result favoured the intervention group for 25 interventions (30%). Some of the recommendations were: use of antiplatelet medication during the 24-hour hospital stay, a study of the left ventricle ejection fraction, total cholesterol and LDL measurements, cardiopulmonary resuscitation, and degree of compliance with EB-CPGs recommendations.

## Discussion

Methodological efforts have been made to develop trustworthy EB-CPGs. However, it still seems that some of these EB-CPGs are far from the reality of the clinical practice ( Institute of Medicine (US) Committee on Standards for Developing Trustworthy Clinical Practice Guidelines, 2011).

It is essential to emphasise the main findings from the outcome analysis. It is surprising that most of the studies did differentiate between the control and experimental groups regarding the improvements with the use of EB-CPGs. The effects of recommendations of the interventions included in the nine RCTs on the areas of health care structure (4%) and patient outcomes were the least studied (32%). This fact could lead us to assume that researchers have given more importance to the evaluation of using (or not using) the recommendations in the area of the process, instead of their direct impact on the patient health.

The changes observed in the patient progression were generally modest. In the six studies evaluating patient outcomes (27 interventions), four of them reported a positive result for seven measurements (26%) of the interventions. For example, the study by Ellis *et al*. (2000) measured the difference in the average change of total cholesterol (mg/dl) and reported a decrease of 7.4 mg/dl in favour of the intervention. It is essential to mention that these measures were mostly surrogate variables. Only one study reported variables for clinical results ( Tierney *et al*., 2003), with 13 interventions, but found no meaningful differences in the results between the groups. 

Most of the studies (eight) reported results in the process area and showed favourable results for the intervention in six studies with 16 measurements. This fact reflects the reluctance of health care providers to use EB-CPGs and means that only 30% of these interventions followed the recommendations given by the EB-CPGs.

Our findings in this review coincide with some of those captured by Gagliardi *et al*. (2012) and Kastner *et al*. (2015) most of the studies included in this review used multiple strategies to implement the EB-CPGs (electronic notifications in a digital file, cell phone applications, letters, phone call memos, printed material), and the authors do not tailor to every recommendation (the implementation strategy is usually the same for every recommendation within a EB-CPG). Cosby (2006) formulates very clearly that although clinicians have the new evidence at hand, using it means a change in the behaviour and habits of the clinical management of patients that is complex to achieve unless appropriate strategies are applied. A more specific approach, based on the results of the analysis of the obstacles hindering the adoption of every recommendation separately, might improve the use and effect of the recommendations in practice.

## Conclusions

There is an imbalance between the number of EB-CPGs developed and the number of high-quality studies evaluating their effectiveness. After analysing many studies, we can have one more hypothesis for further research for more light upon those undiscovered variables that might interfere with the use of the EB-CPGs. Therefore, more studies of good quality are still needed.

The variation in the effects of the recommendations of the EB-CPGs suggests that it would be useful to focus on the analysis of the adherence limitations, as well as on designing implementation strategies by adapting every recommendation, instead of considering the EB-CPGs as a whole. Further research is still needed to determine which factors related to the EB-CPGs and their specific recommendations are essential to predict the use of EB-CPGs, and thus achieve better patient outcomes.

### Implications for practice

The initial objective of this review was to strengthen the development programs for EB-CPG by evaluating their effects on the quality of health care and to give reliable evidence to sustain the decision-making process related to the construction of EB-CPGs in medium- and low-income countries. Even the research evidence is not strong enough to support the EB-CPGs as a tool to improve the practice for a better quality of care, the results of this review need to be interpreted with caution. Definitely. It seems the standard application used so far must be reviewed and must incorporate new psychosocial strategies oriented toward driving change in clinical practice and the doctor-patient relationship We need reaching the right hands at the right time. It is necessary to emphasise that the standard CPG implementation used so far must be reviewed, and must incorporate new psychosocial strategies oriented toward driving change in clinical practice and the doctor-patient relationships.

For an adequate implementation of a EB-CPG, it is necessary to take into account the possible costs, risks, and benefits that will be necessary to know the expected results precisely. The health systems that build EB-CPGs must maximise their efforts to get health care personnel to follow the recommendations of the EB-CPGs and make an effort to evaluate their impact.

### Implications for research

This is the first of series of three systematic reviews on the effects of Evidence-Based Clinical Practice Guidelines (EB-CPGs) on health care quality improvements. The second RD will cover Breast Cancer and the third child and maternal health. We created an interactive https://lnkd.in/dwTxaD8 LIVE GUIDELINES program following the CDC https://lnkd.in/d5r4z6m

Since research in this field is so new, and the results of this study were not conclusive, more research is needed to evaluate the change that EB-CPGs could make to the quality of health care, emphasising the less studied areas, such as the structure of health care services and patient outcomes.

## Data availability

### Underlying data

All data underlying the results are available as part of the article and no additional source data are required.

### Extended data

Open Science Framework: Effects of Evidence-Based Clinical Practice Guidelines in cardiovascular health care quality improvements- A Systematic Review. https://doi.org/10.17605/OSF.IO/9A5FM ( Ramirez *et al*., 2019).

This project contains the following extended data:

- Appendix 1 Advanced Search Strategy
- Appendix 2 List of studies selected after screening and assessing the full text
- Appendix 3 Characteristics of included studies English version
- Some electronic data extraction forms

### Reporting guidelines

Open Science Framework: PRISMA checklist for “Effects of evidence-based clinical practice guidelines in cardiovascular health care quality improvements: A systematic review”. https://doi.org/10.17605/OSF.IO/9A5FM ( Ramirez *et al*., 2019).

## Author information

Anggie Ramírez-Morera and Juan Carlos Vazquez are PhD candidates Program in Biomedical Research Methodology and Public Health, Universitat Autònoma de Barcelona

## References

[ref-31] Alonso-Coello P Irfan A Solà I: The quality of clinical practice guidelines over the last two decades: a systematic review of guideline appraisal studies. *Qual Saf Health Care.* 2010;19(6):e58. 10.1136/qshc.2010.042077 21127089

[ref-1] Beaulieu MD Brophy J Jacques A: Drug treatment of stable angina pectoris and mass dissemination of therapeutic guidelines: a randomized controlled trial. *QJM.* 2004;97(1):21–31. 10.1093/qjmed/hch006 14702508

[ref-2] Berner ES Baker CS Funkhouser E: Do local opinion leaders augment hospital quality improvement efforts? A randomized trial to promote adherence to unstable angina guidelines. *Med Care.* 2003;41(3):420–31. 10.1097/01.MLR.0000052977.24246.38 12618645

[ref-32] Cosby JL: Improving patient care: the implementation of change in clinical practice. *Qual Saf Health Care.* 2006;15(6):447 10.1136/qshc.2005.016824

[ref-3] Donabedian A: The quality of care. How can it be assessed? *JAMA.* 1988;260(12):1743–1748. 10.1001/jama.1988.03410120089033 3045356

[ref-4] Ellis SL Carter BL Malone DC: Clinical and economic impact of ambulatory care clinical pharmacists in management of dyslipidemia in older adults: the IMPROVE study. Impact of Managed Pharmaceutical Care on Resource Utilization and Outcomes in Veterans Affairs Medical Centers. *Pharmacotherapy.* 2000;20(12):1508–1516. 10.1592/phco.20.19.1508.34852 11130223

[ref-33] Gagliardi AR Brouwers MC Bhattacharyya OK: The guideline implementability research and application network (GIRAnet): an international collaborative to support knowledge exchange: study protocol. *Implement Sci.* 2012;7:26. 10.1186/1748-5908-7-26 22471937 PMC3338081

[ref-34] Glasziou P Ogrinc G Goodman S: Can evidence-based medicine and clinical quality improvement learn from each other? *BMJ Qual Saf.* 2011;20 Suppl 1:i13–17. 10.1136/bmjqs.2010.046524 21450763 PMC3066698

[ref-35] Grimshaw JM Russell IT: Effect of clinical guidelines on medical practice: a systematic review of rigorous evaluations. *Lancet.* 1993;342(8883):1317–1322. 10.1016/0140-6736(93)92244-n 7901634

[ref-5] Guadagnoli E Normand SL DiSalvo TG: Effects of treatment recommendations and specialist intervention on care provided by primary care physicians to patients with myocardial infarction or heart failure. *Am J Med.* 2004;117(6):371–9. 10.1016/j.amjmed.2004.04.013 15380493

[ref-36] Guyatt G, Evidence-Based Medicine Working Group: Evidence-based medicine. A new approach to teaching the practice of medicine. *JAMA.* 1992;268(17):2420–2425. 10.1001/jama.1992.03490170092032 1404801

[ref-6] Hand WR Bridges KH Stiegler MP: Effect of a cognitive aid on adherence to perioperative assessment and management guidelines for the cardiac evaluation of noncardiac surgical patients. *Anesthesiology.* 2014;120(6):1339–49, quiz 1349-53. 10.1097/ALN.0000000000000251 24705442 PMC4108481

[ref-7] Higgins JPT Green S(editors): Cochrane Handbook for Systematic Reviews of Interventions Version 5.1.0 [updated March 2011]. Barcelona: The Cochrane Collaboration.2012; [Accessed February 21, 2019]. Reference Source

[ref-8] Institute of Medicine (US) Committee on Standards for Developing Trustworthy Clinical Practice Guidelines: Clinical practice guidelines we can trust R. Graham *et al*., eds., Washington (DC): National Academies Press (US).2011. 10.17226/13058 24983061

[ref-9] Jäntti H Kuisma M Uusaro A: The effects of changes to the ERC resuscitation guidelines on no flow time and cardiopulmonary resuscitation quality: a randomised controlled study on manikins. *Resuscitation.* 2007;75(2):338–344. 10.1016/j.resuscitation.2007.05.006 17628319

[ref-37] Kastner M Bhattacharyya O Hayden L: Guideline uptake is influenced by six implementability domains for creating and communicating guidelines: a realist review. *J Clin Epidemiol.* 2015;68(5):498–509. 10.1016/j.jclinepi.2014.12.013 25684154

[ref-10] Kiessling A Henriksson P: Efficacy of case method learning in general practice for secondary prevention in patients with coronary artery disease: randomised controlled study. *BMJ.* 2002;325(7369):877–880. 10.1136/bmj.325.7369.877 12386042 PMC129638

[ref-38] Lugtenberg M Burgers JS Westert GP: Effects of evidence-based clinical practice guidelines on quality of care: a systematic review. *Qual Saf Health Care.* 2009;18(5):385–392. 10.1136/qshc.2008.028043 19812102

[ref-39] Moore L Lavoie A Bourgeois G: Donabedian’s structure-process-outcome quality of care model: Validation in an integrated trauma system. *J Trauma Acute Care Surg.* 2015;78(6):1168–75. 10.1097/TA.0000000000000663 26151519

[ref-11] Ramirez A Tristan M Vazquez JC: Effects of Evidence-Based Clinical Practice Guidelines in cardiovascular health care quality improvements- A Systematic Review. OSF.2019 10.17605/OSF.IO/9A5FM PMC6790909 31656589

[ref-40] Sackett DL Rosenberg WM Gray JA: Evidence based medicine: what it is and what it isn’t. *BMJ.* 1996;312(7023):71–72. 10.1136/bmj.312.7023.71 8555924 PMC2349778

[ref-12] Tierney WM Overhage JM Murray MD: Effects of computerized guidelines for managing heart disease in primary care. *J Gen Intern Med.* 2003;18(12):967–976. 10.1111/j.1525-1497.2003.30635.x 14687254 PMC1494965

[ref-13] Tsuyuki RT Houle SK Charrois TL: Randomized Trial of the Effect of Pharmacist Prescribing on Improving Blood Pressure in the Community: The Alberta Clinical Trial in Optimizing Hypertension (RxACTION). *Circulation.* 2015;132(2):93–100. 10.1161/CIRCULATIONAHA.115.015464 26063762

[ref-41] Worrall G Chaulk P Freake D: The effects of clinical practice guidelines on patient outcomes in primary care: a systematic review. *CMAJ.* 1997;156(12):1705–1712. 9220922 PMC1227585

