# Supplementary material for: Effects of evidence-based clinical practice guidelines in cardiovascular health care quality improvements: A systematic review
Source: F1000Res. 2019 Oct 14;8:1041. Originally published 2019 Jul 10. [Version 3] doi: 10.12688/f1000research.18865.3 (PMC6790909; doi:10.12688/f1000research.18865.3)
Supplement: Peer review history

# Peer review: Reviewer response for version 2

**DOI:** 10.5256/f1000research.22588.r54141

**Published:** 2019-10-10

**Authors:** Ignacio Marín León

**Affiliations:** 1 Fundación Enebro, Seville, Spain

It is OK, the new version of the article clearly improve the original one and include the reviewers suggestions.

I confirm that I have read this submission and believe that I have an appropriate level of expertise to confirm that it is of an acceptable scientific standard.

# Peer review: Reviewer response for version 1

**DOI:** 10.5256/f1000research.20676.r51028

**Published:** 2019-08-22

**Authors:** Ignacio Marín León

**Affiliations:** 1 Fundación Enebro, Seville, Spain

Thank you for the invitation to review this well-conducted systematic review.

 The study under review is very interesting and necessary for the Clinical Practice Guidelines (CPGs) community and for the implementation science and healthcare quality communities.

 I would like to congratulate the authors for this difficult challenge with some minor comments to contribute to improve the content and message of this necessary study

**ABSTRACT: Conclusion**

- *"There are probably still undiscovered variables that interfere with the use of the CPGs"*. 
  That could not be a conclusion from the study, given that the aim of the work was not to study factors and conditions that facilitates the implementation of CPG neither the conditions to change professional behavior. No data on these issues are reported, then no conclusion could be provided.
  A coherent conclusion could be the need to improve and systematize this kind of implementation studies. 

***Introduction: **pag 3, para 4, left column*

- *"After an exhaustive search, only two systematic reviews (SR) we found on this topic,"*
  It is needed to provide both references

**RESULTS:** *pag 3, 4 and figure 1. Study identification and selection.*

- *"Of these, we selected 96 studies after assessing the full texts".*
  It is needed, in order to strengthen the transparency and reproducibility of the work, to display in an extended data appendix, the full reference list of rejected originals and reasons to be rejected (96 found minus 9 selected = 87 rejected). It should be clarified what is meant in the figure 1 tab "# 87 of studies to include in other analyzes". 

*Pag 4, left column, para 3, rows 4-6: *

- *"When assessing the full-text articles, we found that many were cluster trials"*
  There is no clear reasons to reject RCT clusters studies, when the intervention could be randomized to cluster offices, and the outcomes effect measured at individual patient level. It is a well-accepted methodology in implementation science, given that can control for bias and non-measured variables. (Please see 2 references provided: Eccles and Romero). ^1^^,^^2^

*Pag 5, Table 1, Rows 2-4 and Column 5: *

- *"Certainty of the evidence (GRADE)"*
  It is difficult to understand why the certainty of evidence for the outcomes "time without flow" and "delay to start CPR" are classified differently when, as displayed in the explanations, the bias and systematic errors are the same, and there is not a loss of patients to follow up. It is true that both effects measure score different statistical significance, but with data provided the readers feel with the same certainty (either moderate or low) that one effect is significantly different from the control group, and for the other outcomes (delay) there are not differences with the control group.

*Pag 6, Table 2, Rows 2-4 and Column 5: *

- *"Certainty of the evidence (GRADE)"*
  The same reasoning for the difficulty to understand why are classified differently the certainty of evidence for the outcomes "LDL cholesterol" and "Total cholesterol".

**Discussion:**** *Pag 7, para 2, rows 7-13 *

- *"This fact could lead us to assume that researchers have given more importance to the evaluation of using (or not using) the recommendations in the area of the process, instead of their direct impact on the patient health".*
  Another explanation could be to emphasize that it use to be easier to measure process of care than patient health outcomes. These health outcomes take a longer period of observations than to measure how professionals adopt CPG recommendations. That can explain the "preferences" of researchers. 

**Conclusions**

- The same considerations exposed in the abstract regarding the sentences *"There are probably still undiscovered variables that interfere with the use of the CPGs"*. 
  That could not be a conclusion from the study, given that was not the aim of the work to study factors and conditions that facilitates the implementation of CPG neither the conditions to change professional behavior. No data on these issues are reported, them no conclusion could be provided.
  A coherent conclusion could be the needs to improve and systematize these kinds of implementation studies. 

I confirm that I have read this submission and believe that I have an appropriate level of expertise to confirm that it is of an acceptable scientific standard, however I have significant reservations, as outlined above.

## References

[1] : Research designs for studies evaluating the effectiveness of change and improvement strategies. *Qual Saf Health Care*.2003;12(1) : 10.1136/qhc.12.1.47 47-52 10.1136/qhc.12.1.47 12571345PMC1743658

[2] : Efectividad de la implantación de una guía clínica sobre la angina inestable mediante una estrategia multifactorial. Ensayo clínico aleatorizado en grupos. *Revista Española de Cardiología*.2005;58(6) : 10.1157/13076416 640-648 10.1157/13076416 15970119

# Response

**Published:** 2019-09-05

**Authors:** Anggie Ramirez

**Dear Ignacio Marín León**

 Thank you for your Review Report

 This response was concomitant between Dr. Ramirez and Dr. Tristan, part of the authors of this systematic review.

  APPROVED WITH RESERVATIONS

 Thank you for the invitation to review this well-conducted systematic review.

 The study under review is very interesting and necessary for the Clinical Practice Guidelines (CPGs) community and for the implementation of science and healthcare quality communities.

 I would like to congratulate the authors for this difficult challenge with some minor comments to contribute to improving the content and message of this necessary study

**#1 ABSTRACT: Conclusion**

- *"There are probably still undiscovered variables that interfere with the use of the CPGs"*. 
  That could not be a conclusion from the study, given that the aim of the work was not to study factors and conditions that facilitates the implementation of CPG neither the conditions to change professional behavior. No data on these issues are reported, then no conclusion could be provided.
  A coherent conclusion could be the need to improve and systematize this kind of implementation studies. 

**Answer: **We change the text for a better understanding of our meaning. After analyzing many studies, we can have one more hypothesis for further research for more light upon those undiscovered variables that might interfere with the use of the CPGs.

***#2 Introduction:*** *pag 3, para 4, left column*

- *"After an exhaustive search, only two systematic reviews (SR) we found on this topic,"*
  It is needed to provide both references

**Answer: **We agree and change for: After an exhaustive search, only two systematic reviews (SR) we found on this topic (Worrall, 1997; Lugtenberg, 2009). Worral (1997) only analysed patient outcomes missing two of the proposed Donadebian Model. Lugtemberg 2009 used the full Donadebian (1988)  model only including studies from The Netherlands.

**#3 RESULTS:** *pag 3, 4 and figure 1. Study identification and selection.*

- *"Of these, we selected 96 studies after assessing the full texts".*
  It is needed, in order to strengthen the transparency and reproducibility of the work, to display in an extended data appendix, the full reference list of rejected originals and reasons to be rejected (96 found minus 9 selected = 87 rejected). It should be clarified what is meant in the figure 1 tab "# 87 of studies to include in other analyses". 

**Answer:** We agree . The Appendix 2: List of studies selected after screening and assessing the full text is added at OSF Home, the link is: https://osf.io/9a5fm/?view_only=0ec7b68ed1004bf6b1aa74a19ed1912d

 We did not reject the 87 publications, not analyzed. This SR is the first of series that has three systematic reviews. This two new ones will include the topics breast cancer, type 2 diabetes and others.

***#4*** *Pag 4, left column, para 3, rows 4-6: *

- *"When assessing the full-text articles, we found that many were cluster trials"*
  There is no clear reasons to reject RCT clusters studies, when the intervention could be randomized to cluster offices, and the outcomes effect measured at individual patient level. It is a well-accepted methodology in implementation science, given that can control for bias and non-measured variables. (Please see 2 references provided: Eccles and Romero). ^1^ ^,^ ^2^

**Answer: **The authors decide when preparing the protocol to include only RCT.

*#5 Pag 5, Table 1, Rows 2-4 and Column 5: *

- *"Certainty of the evidence (GRADE)"*

It is difficult to understand why the certainty of evidence for the outcomes "time without flow" and "delay to start CPR" are classified differently when, as displayed in the explanations, the bias and systematic errors are the same, and there is not a loss of patients to follow up. It is true that both effects measure score different statistical significance, but with data provided the readers feel with the same certainty (either moderate or low) that one effect is significantly different from the control group, and for the other outcomes (delay) there are not differences with the control group.

**Answer: **Please check the note explanations both have different statistical significance due to this fact the certainty of evidence is downgraded for “Delay to start CPR (s)”.

*#6 Pag 6, Table 2, Rows 2-4 and Column 5: *

- *"Certainty of the evidence (GRADE)"*

The same reasoning for the difficulty to understand why are classified differently the certainty of evidence for the outcomes "LDL cholesterol" and "Total cholesterol".

**Answer: **Please check the note explanations both have different statistical significance due to this fact the certainty of evidence is downgraded for “Difference in percent change in total cholesterol at 2 years [Mean (mmol / L)]”.

**#7 Discussion:** *Pag 7, para 2, rows 7-13 *

- *"This fact could lead us to assume that researchers have given more importance to the evaluation of using (or not using) the recommendations in the area of the process, instead of their direct impact on the patient health".*
  Another explanation could be to emphasize that it use to be easier to measure process of care than patient health outcomes. These health outcomes take a longer period of observations than to measure how professionals adopt CPG recommendations. That can explain the "preferences" of researchers. 

**Answer: **You are right.

**#10 Conclusions**

- The same considerations exposed in the abstract regarding the sentences *"There are probably still undiscovered variables that interfere with the use of the CPGs"*.

That could not be a conclusion from the study, given that was not the aim of the work to study factors and conditions that facilitates the implementation of CPG neither the conditions to change professional behavior. No data on these issues are reported, them no conclusion could be provided.

 A coherent conclusion could be the needs to improve and systematize these kinds of implementation studies. 

**Answer: **We change the text for a better understanding of our meaning. After analyzing many studies, we can have one more hypothesis for further research for more light upon those undiscovered variables that might interfere with the use of the CPGs.

# Peer review: Reviewer response for version 1

**DOI:** 10.5256/f1000research.20676.r51034

**Published:** 2019-07-24

**Authors:** Yasser Sami Abdel Dayem Amer

**Affiliations:** 1 Clinical Practice Guidelines Unit, Quality Management Department, King Saud University Medical City, Riyadh, Saudi Arabia; 2 Alexandria Center for Evidence-Based Clinical Practice Guidelines, Alexandria University, Alexandria, Egypt

Thank you for the invitation to review this well-conducted systematic review.

 The study under review is very interesting for the evidence-based clinical practice guidelines (CPGs)- and for the healthcare quality and safety- research communities.

 Moreover, I would like to congratulate the authors for this work with some minor comments with the aim of improving the content and message of this distinguished study.

**Abstract:**

  

1. I recommend replacing the phrase 'results in patients' with *' patient outcomes' *as it is more commonly used in the field of quality and safety in healthcare. Additionally, the authors have used it for the rest of the MS.
**Introduction:**
2. I would recommend adding ***recent *defi*nitions of both CPGs and healthcare quality (HCQ)*** as nowadays clinicians are getting increasingly interested in the fields of evidence-based medicine (EBM) and HCQ either from a research perspective or through engagement in their respective healthcare organizational accreditation. The HCQ definition and description of EB-CPGs are already in the PROSPERO protocol, please include them here as well.
*Additional relevant studies to cite here:*
  (i) about the relation between EBM and HCQ:
  Glasziou P, Ogrinc G, Goodman S. Can evidence-based medicine and clinical quality improvement learn from each other?. *BMJ Qual Saf*. 2011;20 Suppl 1(Suppl_1):i13–i17. doi:10.1136/bmjqs.2010.046524 ^1^
  (ii) an earlier similar key review:
  Grimshaw J, Russell I: Effect of clinical guidelines on medical practice: a systematic review of rigorous evaluations. *The Lancet*. 1993; **342** (8883): 1317-1322 ^2^
  page 3, left column, para 4:
3. '..After an exhaustive.....only two systematic reviews *(SRs)*....., but *' these SRs' *did not....'
  Please add citations for these two references (SRs).
4. '...and international perspective together.' What do you mean by an 'international perspective'; of what; (i.e. of CPGs, of healthcare quality, or of cardiovascular healthcare?), please clarify or re-write.
  page 3, right column, para 4:
5. Regarding the data extraction modified and translated from the EPOC checklist, was it validated and if not, why the authors felt they did not need to? Additionally, please provide the readers who would be interested in the replication of this research with this tool as an online supplementary here.
  It was indeed mentioned with a link in the PROSPERO protocol ( https://fs20.formsite.com/mtristan/form41/index.html, attached on Appendix 3) but the link did not work, giving an error!
  Moreover, I checked the (Extended data) links in the OSF Home but could not retrieve it.
6. Please delete the duplicated sentence; 'We used a standardized digital ..........and analysis'.
  page 3, right column, para 7:
7. The #87 studies that were planned to be included in another analysis were not mentioned here since the final included were 9 rather than 96 studies. Please elaborate on this in this location.
8. Figure 1. PRISMA flow chart:
  #87 of studies to include in other *' analyses'.*
  Tables 1 and 2.: the GRADE quality assessments and the explanations for the certainty of evidence were very well conducted and written.
9. Table 1: please, if feasible, replace the question in the GRADE SoF table; '.....the results for cardiopulmonary resuscitation?' with '.....the *outcomes* for cardiopulmonary resuscitation?'
  page 7, left column para 1:
10. Once more, please replace 'patient results' with ' *patient outcomes'.*
  page 7, left column, para4:
  Discussion:
11. '..However, it still seems that some of *these* CPGs are...' 
  page 7, right column, para 2:
12. As part of discussing 'multiple strategies to implement the CPGs', I would recommend adding insights and citing these relevant keynote articles that discuss multi-faceted CPGI strategies and their importance:
  (i) Grol R, Wensing M. Improving patient care: the implementation of change in health care (in Dutch: Implementatie: effective verbetering van de patiëntenzorg). Amsterdam: Reed Business Education; 2013.
  (ii) Kastner M, Bhattacharyya O, Hayden L, et al. Guideline uptake is influenced by six implementability domains for creating and communicating guidelines: a realist review. J Clin Epidemiol. 2015;68(5):498–509. doi: 10.1016/j.jclinepi.2014.12.013. ^3^
  (iii) Gagliardi A, Brouwers M, Bhattacharyya O, et al. A framework of the desirable features of guideline implementation tools (GItools): Delphi survey and assessment of GItools. Implement Sci. 2014;9:98. doi: 10.1186/s13012-014-0098-8 ^4^
  (iv) Gagliardi A, Brouwers M, Palda V, Lemieux-Charles L, Grimshaw J. How can we improve guideline use? A conceptual framework of implementability. Implement Sci. 2011;6:26. doi: 10.1186/1748-5908-6-26. ^5^
  page 7, right column, para 4:
13. '...... as well as on designing implementation strategies adapt every recommendation...'
  Do you mean "designing implementation strategies AND adapt every recommendation....if so add a comma ',' " or "designing implementation strategies (by) adapting every recommendation".
  Additionally, I suggest to add:
*'* *....initiate a pilot implementation of specific recommendation(s) *instead of considering *implementation of* the CPGs as a whole'.
  page 7, right column, para 5:
14. Implications for practice The initial objective of this review was to strengthen the development programs for *CPGs (plural)*
15. The results of this review must need to be interpreted with caution.
  It reads better either (the results of this review *must be* interpreted with caution.) or (the results of this review *need to be* interpreted with caution.).
16. 'It is necessary to emphasise that the standard *CPG* implementation used so...'
  page 7, right column, para 6:
17. 'For an adequate implementation of a CPG, it is necessary to take into account the possible costs, ** *risks, and* **benefits

Finally, I would like to congratulate the authors once more on this work and invite all interested researchers to conduct similar research using this robust methodology to study the impact of EB-CPG implementation on all different healthcare specialties in addition to their effects on the interdisciplinary workflows and transitions of care between different specialties and services in healthcare.

I confirm that I have read this submission and believe that I have an appropriate level of expertise to confirm that it is of an acceptable scientific standard.

## References

[1] : Can evidence-based medicine and clinical quality improvement learn from each other?. *BMJ Qual Saf*.2011;20 Suppl 1: 10.1136/bmjqs.2010.046524 i13-17 10.1136/bmjqs.2010.046524 21450763PMC3066698

[2] : Effect of clinical guidelines on medical practice: a systematic review of rigorous evaluations. *Lancet*.1993;342(8883) : 10.1016/0140-6736(93)92244-n 1317-22 10.1016/0140-6736(93)92244-n 7901634

[3] : Guideline uptake is influenced by six implementability domains for creating and communicating guidelines: a realist review. *J Clin Epidemiol*.2015;68(5) : 10.1016/j.jclinepi.2014.12.013 498-509 10.1016/j.jclinepi.2014.12.013 25684154

[4] : A framework of the desirable features of guideline implementation tools (GItools): Delphi survey and assessment of GItools. *Implement Sci*.2014;9: 10.1186/s13012-014-0098-8 98 10.1186/s13012-014-0098-8 25091091PMC4244063

[5] : How can we improve guideline use? A conceptual framework of implementability. *Implement Sci*.2011;6: 10.1186/1748-5908-6-26 26 10.1186/1748-5908-6-26 21426574PMC3072935

# Response

**Published:** 2019-09-05

**Authors:** Anggie Ramirez

**Dear Yasser Sami Abdel Dayem Amer**

Thank you for your Review Report

This response was concomitant between Dr. Ramirez and Dr. Tristan, part of the authors of this systematic review.

Thank you for the invitation to review this well-conducted systematic review.

The study under review is very interesting for the evidence-based clinical practice guidelines (CPGs)- and for the healthcare quality and safety- research communities.

Moreover, I would like to congratulate the authors for this work with some minor comments with the aim of improving the content and message of this distinguished study.

**Abstract:**

1. I recommend replacing the phrase 'results in patients' with *' patient outcomes' *as it is more commonly used in the field of quality and safety in healthcare. Additionally, the authors have used it for the rest of the MS.

**Answer: **We agree and we will make the change.

**Introduction:** 

1. I would recommend adding ***recent definitions of both CPGs and healthcare quality (HCQ)*** as nowadays clinicians are getting increasingly interested in the fields of evidence-based medicine (EBM) and HCQ either from a research perspective or through engagement in their respective healthcare organizational accreditation. The HCQ definition and description of EB-CPGs are already in the PROSPERO protocol, please include them here as well. *Additional relevant studies to cite here:*(i) about the relation between EBM and HCQ:Glasziou P, Ogrinc G, Goodman S. Can evidence-based medicine and clinical quality improvement learn from each other?. *BMJ Qual Saf*. 2011;20 Suppl 1(Suppl_1):i13–i17. doi:10.1136/bmjqs.2010.046524 ^1^(ii) an earlier similar key review:Grimshaw J, Russell I: Effect of clinical guidelines on medical practice: a systematic review of rigorous evaluations. *The Lancet*. 1993; **342** (8883): 1317-1322 ^2^ **Answer: **We agree and we will make the change CPGs for EB-GPGs and added the recommended references the are very useful.

page 3, left column, para 4:

1. '..After an exhaustive.....only two systematic reviews *(SRs)*....., but *' these SRs' *did not....'Please add citations for these two references (SRs).

**Answer: **We agree, and we will make the change.

 

1. '...and international perspective together.' What do you mean by an 'international perspective'; of what; (i.e. of CPGs, of healthcare quality, or of cardiovascular healthcare?), please clarify or re-write.

**Answer: **We agree, and we will make the change for: after an exhaustive search, only two systematic reviews (SR) we found on this topic (Worrall 1997, Lugtenberg 2009). Worral (1997) only analysed patient outcomes missing two of the proposed Donadebian Model. Lugtemberg 2009 used the full Donadebian (1988)  model only including studies from The Netherlands.

page 3, right column, para 4:

1. Regarding the data extraction modified and translated from the EPOC checklist, was it validated and if not, why the authors felt they did not need to? Additionally, please provide the readers who would be interested in the replication of this research with this tool as an online supplementary here.It was indeed mentioned with a link in the PROSPERO protocol ( https://fs20.formsite.com/mtristan/form41/index.html, attached on Appendix 3) but the link did not work, giving an error!Moreover, I checked the (Extended data) links in the OSF Home but could not retrieve it.

**Answer: We agree, and we will make the change. **The Some electronic data extraction forms the link is not available now it is a temporary link during the active time of the survey period in the form site server.  Now some results of the on line survey are  available  in this link https://osf.io/9a5fm/?view_only=0ec7b68ed1004bf6b1aa74a19ed1912d as  Some electronic data extraction forms/Short version of EPOC Check list.docx. The link in the OSF Home is working.

 

1. Please delete the duplicated sentence; 'We used a standardized digital ..........and analysis'. **Answer: **We agree, and we will make the change

 

page 3, right column, para 7:

1. The #87 studies that were planned to be included in another analysis were not mentioned here since the final included were 9 rather than 96 studies. Please elaborate on this in this location.

**Answer: **We agree, and we will make the change. And we add the following:

When the first version of the protocol of this Systematic Review was initiated, consider the inclusion of several clinical topics in a single systematic review. We decided to create a series of three systematic reviews on different clinical topics: Cardiovascular Health, Breast Cancer and Child and Mother Health. The initial searches included all those topics with the same inclusion criteria described. After removing duplicates, the search produced 4279 potential studies. After screening by title and abstract, 4051 were excluded. After the full-text evaluation, 96 studies of all clinical subjects were selected. For this SR, only nine studies covering the cardiovascular issue were included.

This is the first of series of three systematic reviews on the effects of Evidence-Based Clinical Practice  Guidelines (EB-CPGs) on health care quality improvements. The second RD  will cover Breast Cancer and the third child and maternal health.  We created an interactive https://lnkd.in/dwTxaD8 LIVE GUIDELINES program following the CDC https://lnkd.in/d5r4z6m

 

1. Figure 1. PRISMA flow chart: #87 of studies to include in other *' analyses'.*

**Answer: **We agree, and we will make the change

Tables 1 and 2.: the GRADE quality assessments and the explanations for the certainty of evidence were very well conducted and written.

1. Table 1: please, if feasible, replace the question in the GRADE SoF table; '.....the results for cardiopulmonary resuscitation?' with '.....the *outcomes* for cardiopulmonary resuscitation?'

**Answer: **We agree, and we will make the change

page 7, left column para 1:

1. Once more, please replace 'patient results' with ' *patient outcomes'.*

**Answer: **We agree, and we will make the change

page 7, left column, para4:Discussion:

1. '..However, it still seems that some of *these* CPGs are...' 

**Answer: **We agree, and we will make the change

page 7, right column, para 2:

1. As part of discussing 'multiple strategies to implement the CPGs', I would recommend adding insights and citing these relevant keynote articles that discuss multi-faceted CPGI strategies and their importance:(i) Grol R, Wensing M. Improving patient care: the implementation of change in health care (in Dutch: Implementatie: effective verbetering van de patiëntenzorg). Amsterdam: Reed Business Education; 2013.

(ii) Kastner M, Bhattacharyya O, Hayden L, et al. Guideline uptake is influenced by six implementability domains for creating and communicating guidelines: a realist review. J Clin Epidemiol. 2015;68(5):498–509. doi: 10.1016/j.jclinepi.2014.12.013. ^3^

(iii) Gagliardi A, Brouwers M, Bhattacharyya O, et al. A framework of the desirable features of guideline implementation tools (GItools): Delphi survey and assessment of GItools. Implement Sci. 2014;9:98. doi: 10.1186/s13012-014-0098-8 ^4^

(iv) Gagliardi A, Brouwers M, Palda V, Lemieux-Charles L, Grimshaw J. How can we improve guideline use? A conceptual framework of implementability. Implement Sci. 2011;6:26. doi: 10.1186/1748-5908-6-26. ^5^

**Answer: **Thank you very much for the recommended references. We agree that they will give strength to the implementation aspect, one of the most sensitive components for the use of CPG.

In relation to the reference  Gagliardi 2011, We will not take it in the article because the Research Implementation Guide and Application Network (GiRAnet), is not active and in its link http://www.cebhc.co.za/guideline- implementability-research -y-application-network-giranet-2 /, one link does not work and the other is restricted access.

We had access to another article by the same author of 2012, which we share ideas that we have already implemented and this study is in protocol: Gagliardi, A. R., Brouwers, M. C., & Bhattacharyya, O. K. (2012). The guideline implementability research and application network (GIRAnet): an international collaborative to support knowledge exchange: study protocol. Implementation Science, 7, 26. https://doi.org/10.1186/1748-5908-7-26

The new paragraph reads as follows:

Our findings in this review coincide with some of those captured by Gagliardi (2012) and Kastner (2015) most of the studies included in this review used multiple strategies to implement the EB-CPGs (electronic notifications in a digital file, cell phone applications, letters, phone call memos, printed material), and the authors do not tailor to every recommendation (the implementation strategy is usually the same for every recommendation within a EB-CPG) Cosby (2006) formulates very clearly that although clinicians have the new evidence at hand, using it means a change in the behaviour and habits of the clinical management of patients that is complex to achieve unless appropriate strategies are applied. A more specific approach, based on the results of the analysis of the obstacles hindering the adoption of every recommendation separately, might improve the use and effect of the recommendations in practice.

page 7, right column, para 4:

1. '...... as well as on designing implementation strategies adapt every recommendation...'Do you mean "designing implementation strategies AND adapt every recommendation....if so add a comma ',' " or "designing implementation strategies (by) adapting every recommendation". Additionally, I suggest to add: *' ....initiate a pilot implementation of specific recommendation(s) *instead of considering *implementation of* the CPGs as a whole'.

**Answer:** We partially agree, and we will make the change. We will not mention or suggest of pilot program implementation because most of them majority yield very little or nothing.  

page 7, right column, para 5:

1. Implications for practice The initial objective of this review was to strengthen the development programs for *CPGs (plural)*

**Answer: **We agree, and we will make the change 

 

1. The results of this review must need to be interpreted with caution. It reads better either (the results of this review *must be* interpreted with caution.) or (the results of this review *need to be* interpreted with caution.).

 
**Answer: **We agree, and we will make the change 

 

1. 'It is necessary to emphasise that the standard *CPG* implementation used so...' **Answer: **We agree, and we will make the change 

 

page 7, right column, para 6:

1. 'For an adequate implementation of a CPG, it is necessary to take into account the possible costs, *risks, and *benefits

**Answer: **We agree, and we will make the change
